# Detection of Upper Limb Asymmetries in Athletes According to the Stage of the Season—A Longitudinal Study

**DOI:** 10.3390/ijerph19020849

**Published:** 2022-01-13

**Authors:** Álvaro Velarde-Sotres, Antonio Bores-Cerezal, Marcos Mecías-Calvo, Martín Barcala-Furelos, Silvia Aparicio-Obregón, Julio Calleja-González

**Affiliations:** 1 Facultad de Ciencias de la Salud, Universidad Europea del Atlántico, 39011 Santander, Spain; alvaro.velarde@uneatlantico.es (Á.V.-S.); antonio.bores@uneatlantico.es (A.B.-C.); marcos.mecias@usc.es (M.M.-C.); martin.barcala@uneatlantico.es (M.B.-F.); 2 Departamento de Salud, Universidad Internacional Iberoamericana, Campeche 24560, Mexico; 3 Facultad de Formación del Profesorado, Universidade de Santiago de Compostela, 27001 Lugo, Spain; 4 Facultad de Ciencias Sociales y Humanidades, Universidad Europea del Atlántico, 39011 Santander, Spain; silvia.aparicio@uneatlantico.es; 5 Faculdade de Ciências Sociais e Humanas, Universidade Internacional do Cuanza Bairro Kaluanda, Cuito EN 250, Bié, Angola; 6 Department of Physical Education and Sport, Faculty of Education and Sport, University of the Basque Country (UPV/EHU), 01007 Vitoria, Spain

**Keywords:** functional tests, injury prevention, functional assessment, asymmetries

## Abstract

Sports injuries can affect the performance of athletes. For this reason, functional tests are used for injury assessment and prevention, analyzing physical or physiological imbalances and detecting asymmetries. The main aim of this study was to detect the asymmetries in the upper limbs (right and left arms) in athletes, using the OctoBalance Test (OB), depending on the stage of the season. Two hundred and fifty-two participants (age: 23.33 ± 8.96 years old; height: 178.63 ± 11.12 cm; body mass: 80.28 ± 17.61 kg; body mass index: 24.88 ± 4.58; sports experience: 12.52 ± 6.28 years), practicing different sports (rugby, athletics, football, swimming, handball, triathlon, basketball, hockey, badminton and volleyball), assessed with the OB in medial, superolateral, and inferolateral directions in both arms, in four moments of the season (May 2017, September 2017, February 2018 and May 2018). ANOVA test was used with repeated measures with a *p* ≤ 0.05, for the analysis of the different studied variances. Significant differences were found (*p* = 0.021) in the medial direction of the left arm, between the first (May 2017) and fourth stages (May 2018), with values of 71.02 ± 7.15 cm and 65.03 ± 7.66 cm. From the detection of asymmetries, using the OB to measure, in the medial, superolateral and inferolateral directions, mobility and balance can be assessed. In addition, it is possible to observe functional imbalances, as a risk factor for injury, in each of the stages into which the season is divided, which will help in the prevention of injuries and in the individualization of training.

## 1. Introduction

Recovery and sports injuries can affect the performance of athletes. For this reason, studies and research must be carried out to prevent injuries in sport and improve the performance of athletes, thus contributing new knowledge to science. In addition, materials and methods must be used that allow the correct monitoring and quantification of the load, as well as the study of functional asymmetries.

The study of asymmetries aims to provide information on the presence or absence of imbalances among different body segments at certain times of the season and to establish the most appropriate action protocol for each case.

Functional tests are used for the assessment and prevention of injuries, as they assess the mobility and balance to describe asymmetries or functional imbalance, as injury risk factors [1].

For this reason, it is of great importance to classify high risk factors and the most common and severe injuries, giving priority to sports presenting higher injury risk in respect of frequency and severity [2]. Therefore, some sport modalities are associated with different varieties of injuries. In addition, other possible parameters such as gender, age and activity (practice/competition) are taken into account and will influence the prevalence of injuries [3].

The above-mentioned risk factors can be separated into two groups: modifiable and non-modifiable. Although non-modifiable (gender and age) are of interest, it is similarly significant to study the parameters which are potentially modifiable with daily practice (strength, balance or flexibility) [3].

In the same way, in the context of physical activity and sports, it is essential to carry out an assessment of the athletes, analyzing their evolution or progress on practices, with the aim of reducing the risk of injury [3]. In addition, it is necessary to study the risk of injury of the athletes throughout the season, due to the fact that the training load and physical condition of the athletes is different, and also to carry out an individual control of the athlete, in each of the moments into which the season is divided. To this end, through this research, we have sought to carry out individualized physical monitoring throughout a sports season, carrying out frequent tasks to record information, which will allow us to establish the risk of injury of the athletes analyzed in different periods of the sports season. This is because due to the effects of training, their state will vary throughout the training sessions carried out.

To this end, the articular evaluation Range of Motion (ROM) is used to observe imbalance or asymmetries as risk factors for injury [4,5]. For the development of scientific research, different functional assessment tests can be identified, such as Star Excursion Balance Test (SEBT) [6], and Y-Balance Test (YBT) [7] for the lower body and Upper Quarter Y-Balance Test (UQYBT) [8] and modified-Upper Quarter Y-Balance Test (mUQYBT) [9] for the upper body.

One of the main objectives of the use of these tests is the study of physical or physiological imbalances between different body segments, which can have a negative influence on the athlete’s performance or increase the risk of suffering an injury [5]. These physical or physiological mismatches are called asymmetries [10].

Therefore, it is necessary to achieve a correct balance between the different structures, as well as a reduction in asymmetries [10], using different methods of functional assessment [5] to determine the degree of functional balance between the musculature, as well as an individual approach to data analysis [10].

Given these circumstances, it is necessary to verify the assessment tools, mainly in the upper limbs. One of the tests used for this purpose is the OctoBalance Test (OB) [1]. It includes an octagonal platform device and two measurement systems, which evaluate the mobility in three directions (medial, superolateral and inferolateral), and which could be used by sports scientists to assess injuries and observe asymmetries [11].

The OB Test has been shown previously to be a valid and reliable tool for measuring ROM [11], which allows us to assess mobility and balance, as well as to detect asymmetries or functional imbalances, as an injury risk factor [1].

To date, to the authors’ knowledge, no research or scientific evidence has been found using the OB test for the detection of upper limb asymmetries in different sports, depending on the time of the season.

For this reason, the main objective of this research was to detect asymmetries in the upper limbs (right and left arms) in athletes, using the OB test (direct medial, superolateral and inferolateral), at different times of the sports season.

## 2. Materials and Methods

This research had an experimental design with 252 participants. The OB was utilized as the evaluation tool. The assessment protocol described different movements with the right arm and left arm on the OB platform, analyzing the lengths reached afterward. The evaluation was developed at the laboratory of the European University of the Atlantic, Santander (Spain).

### 2.1. Participants

Two hundred and fifty-two participants: age: 23.33 ± 8.96 years old; height: 178.63 ± 11.12 cm; body mass: 80.28 ± 17.61 kg; body mass index (BMI): 24.88 ± 4.58, and with an experience in sport of 12.52 ± 6.28 years (Table 1), took part in this investigation.

Before the study, all athletes signed their reported consent. This document was compulsory for every athlete and had to be filled out and signed before testing. Inclusion criteria considered that the participants were completely healthy and suitably fit for the test. On contrary, exclusion criteria were: having taken any type of medication; suffering an upper or lower body injury; having practiced any type of training before the test; and suffering any type of illness that would not allow the evaluation protocol to be carried out correctly.

Additionally, the study was evaluated by the Research Ethics Committee of the European Atlantic University, Santander, Spain (code: ID16-IN-022) based on the Helsinki Statement of 2008, updated in Fortaleza, October 2013.

### 2.2. Material

The OB was used as an assessment device (Check your Motion^®^, Albacete, Spain), which involves an octagonal platform and two different measurement systems both effective and scientifically valid [1,11], to assess the functional capacities, identify weaknesses and asymmetries and provide feedback during the practice of the appropriate exercises.

The OB test has been described as a valid, reliable and reproducible device [11], for the measurement of ROM in the upper limb. To the authors’ knowledge, no previous scientific studies have been found using OB testing for the detection of upper limb asymmetries, in different sports, at different times of the sport season.

Finally, for data collection, a record sheet was designed to store the personal data of the athletes, as well as to record each of the measurements obtained in the evaluation.

### 2.3. Protocol

With the aim of evaluating the asymmetries in athletes in the different stages of the season, the OB test was used as an assessment tool, effective and scientifically validated [1,11] to evaluate the functional capacities of athletes, to detect weaknesses and asymmetries and to offer continuous feedback during the practice about correct exercises. For the evaluation, the protocol utilized [11] the validity, reliability and reproducibility of the OB was followed.

The assessment process included the fulfilment of different movements in different directions, medial (Med), superolateral (Sup) and inferolateral (Inf), in both arms, right (R) and left arm (L), platform [11], measuring the length reached afterward, in different stages of the season, May 2017, September 2017, February 2018, and May 2018.

The athletes were familiarized with OB, and had to fill in a form with their personal information: name, surname, age gender, body mass, height, sport practiced, previous injuries and sport experience.

Before evaluation, a standard warm-up was performed, consisting of 3 series of 10 repetitions of squats without external load, push-ups (arms) without load and lunges without load, all of them with a 60″ break between series. Additionally, all participants were up to date with the necessity of avoiding compensations and flexions of the elbow by using correct technique.

The evaluation was carried out with the participants barefoot, in order to guarantee correct test reliability and performance. Three attempts were completed for each direction, medial, superolateral and inferolateral, with each arm having a 10 s passive recovery rest between attempts [1].

It is important to note that attempts were not measured as valid if the mobility or balance failed, lower limbs moved, movements were not performed in the same correct direction and arm movements were not achieved according to the established protocol [9,11]. Finally, when athletes performed a repetition considered null, the test was repeated one more time, until it was completed properly.

### 2.4. Statistical Analysis

Data are shown as a mean (±) standard deviation (SD). Firstly, an analysis was made to check the normal distribution of the data. The Kolmogorov–Smirnov test (>50) was applied to confirm the normality. All variables presented a normal distribution, we decided to use a parametric formula. We set our significance level α at 0.05, with 95% confidence intervals (CI) in all measures.

The Levene statistic was used to test the homogeneity of the variances. In order to estimate the presence of significant differences, an analysis of variance (ANOVA) with repeated measurements was carried out with a *p* ≤ 0.05, for the analysis of the different variables studied of the OB (medial, superolateral and inferolateral direction in both arms) at four moments of the season (May 2017, September 2017, February 2018, and May 2018).

Subsequently, one of the post-hoc tests offering variance analysis was used. In particular, the Scheffé test. The Scheffé test performs multiple joint pair comparisons for all pairs of possible mean combinations, using the F sample distribution. In addition, it can be used to examine all linear combinations of possible mean groups, not just pair comparisons. The Scheffé test was utilized because of its more conservative nature and the characteristics of the sample. Version 25 of Statistics Software package^R^ (SPSS^®^ Inc., Chicago, IL, USA), was used.

## 3. Results

A total of 252 participants took part in the study. Among the 252 participants, 65 were younger than 18 years old, 173 between 18 and 35, and 4 older than 35 years. Related to gender, 210 were men and 42 women. Moreover, 10 different sports were identified; 91 rugby players, 22 athletes, 48 football players, 8 swimmers, 39 handball players, 4 triathletes, 10 basketball players, 9 hockey players, 7 badminton players and 14 volleyball players. Finally, 34 participants were identified as belonging to an endurance sport and 218 participants belonging to a sport of intermittent nature (Table 2).

For the detection of asymmetries, the average of the scores of each of the measures taken at the different moments of the sports season was taken into account. Different variables of the OB were considered: the medial (Med), superolateral (Sup) and inferolateral (Inf) direction of the right arm (R) and left arm (L), in May 2017 (M17), September 2017 (S17), February 2018 (F18) and May 2018 (M18), with the aim of identifying the existence of significant differences with respect to the scores of these variables and the stage of the season.

Significant differences were found (*p* = 0.012), within the analysis of variance (*p* ≤ 0.05), in the medial direction of the left arm. Likewise, significant differences were found (*p* = 0.021) between the first period (May 2017) and the fourth period (May 2018) (Table 3).

No significant differences were observed (*p* = 0.137), within the analysis of variance (*p* ≤ 0.05), in the medial direction of the right arm, in the different moments of the season (Table 4).

The different variables of the OB did not show any significant effect (*p* = 0.117), within the analysis of variance (*p* ≤ 0.05), in the superolateral direction of the right arm, in the different stages of the season (Table 5).

No significant differences were observed (*p* = 0.077), within the assessment of variance (*p* ≤ 0.05), in the superolateral direction of the left arm, in the different stages of the season (Table 6).

The different variables of the OB did not show any significant effect (*p* = 0.160), within the assessment of variance (*p* ≤ 0.05), in the inferolateral direction of the right arm, in the different stages of the season (Table 7).

No significant differences were observed (*p* = 0.347), within the assessment of variance (*p* ≤ 0.05), in the inferolateral direction of the left arm, in the different stages of the season (Table 8).

## 4. Discussion

The main aim of this study was to detect asymmetries in the upper limbs in athletes, depending on the stage of the season.

Through the statistical analyses carried out, significant differences were found (*p* = 0.012), using the analysis of variance (*p* ≤ 0.05), in the medial direction of the left arm. Likewise, significant differences (*p* = 0.021) were found between the first period (May 2017) and the fourth period (May 2018), with values of 71.02 ± 7.15 cm and 65.03 ± 7.66 cm respectively. The presence of significant differences in the medial direction of the left arm show the usefulness of functional tests for the assessment of injuries, evaluating imbalances and joint mobility in order to allow us to determine which one can negatively influence the athlete’s performance or increase the risk of injury [1]. No significant differences were observed in the medial direction of the right arm (*p* = 0.137), in the superolateral direction of the right arm (*p* = 0.117) and of the left arm (*p* = 0.077), nor in the inferolateral direction of the right arm (*p* = 0.160) and the left arm (*p* = 0.347).

Related to the observed asymmetry, the presence of significant differences (*p* = 0.021), between the first period (May 2017) and the fourth period (May 2018), with values of 71.02 ± 7.15 cm and 65.03 ± 7.66 cm, respectively, allows us to identify the changes caused at different times of the season in a limb of the human body. Thus, the results show a decrease in asymmetries in the medial direction of the left arm, in the same period of different seasons (May 2017 and May 2018), as being essential to monitor the athletes, knowing their evolution or progress in training and to decrease injury risk [3].

Therefore, the findings show great objective information that can help prevent the likelihood of injury [5], allow comparison of different stages of the season (May 2017, September 2017, February 2018 and May 2018), as well as to observe asymmetries between limbs, and is necessary for a longitudinal follow-up such as the one carried out [10].

These results, reveal the efficiency of the evaluation the Range of Motion (ROM) in the superolateral, inferolateral and medial direction with OB [11], in order to observe imbalance or asymmetries as a possible risk factor for injury [4,5]. It also shows the importance of using these tests of functional evaluation [1] in different sports, such as rugby, for identifying the asymmetries through the differences in limbs [12]; in football, for identifying the risk factors through the analysis of movement patterns in the preseason [5]; or basketball, as a predictor of lower extremity injury [13], for observing the asymmetries with different functional tests, compared to other tests of performance [1], and to prevent the risk of injury in basketball players [14] by identifying the most common and serious injuries and the main risk factors [3].

This way, the assessment of different types of sports, endurance (34 participants) and sports of intermittent nature (218 participants) shows the necessity of classifying the risk of injury of each sport, as the asymmetries seem to reflect the requirements of each sport [1,5,12]. This risk of injury will not only be defined by the sport or the environmental factor but also the individual characteristics of each participant could influence, in a decisive way [10] the possibility of suffering an injury. Therefore, different sports modalities are related to different types of injuries [3].

Moreover, age (23.33 ± 8.961 years), gender (210 men and 42 women), body mass (80.28 ± 17.61 kg), body mass index (24.88 ± 4.58), experience in the sport (12.52 ± 6.285 years) and type of activity (practice/competition) are parameters which are going to influence the prevalence of injuries [3]. Although non-modifiable risk factors, such as gender and age [14], are of interest, the presence of asymmetries (*p* = 0.021), with values of 71.02 ± 7.15 cm and 65.03 ± 7.66 cm, between different periods (May 2017 and May 2018) show the necessity to analyze potential modifiable parameters related to physical training (strength, balance or flexibility) [3].

Therefore, it can be determined that sport success is based on many and different parameters. Among them, the early identification of the injury and post-interventions that are based on scientific evidence [15,16], are key factors in the prevention and treatment of sports injuries, but always with a reliable and valid assessment, such as that used here.

In addition, optimal recovery after sport or a match is a key factor for sports performance. For proper recovery, the training load should be properly monitored [17] and different recovery protocols and strategies should be used [18,19]. In addition, individual biomarkers should be taken into account to improve health, performance and recovery [20], individual parameters such as sleepiness, recovery profile, stress, soreness and fatigue. Evidence-based recovery will optimize the care and monitoring of athletes [21].

Together with these factors, the identification of asymmetries [10] can have a direct effect on the economic expense which predicts the treatment of the different pathologies derived from the participation in different sports. This way, Junior et al. [16] highlight the direct and proportional relation between the severity of the injury and the medical expense associated to it. Although the economic expenses derived from a sports injury are not the only ones to be taken into account [16], they are the main ones at the time to assess the process injury-recovery purely in an economic way. Therefore, it is necessary to point out that there should be a significant investment in the prevention of these situations, with this type of evaluation.

Therefore, it is necessary to achieve a correct balance between the different structures, as well as a reduction in asymmetries, using different methods of functional assessment to determine the degree of functional balance between the musculature, as well as an individual approach to data analysis [10].

Some potential limitations such as sample, age, gender, type of sport through the season frequently happens in these studies. On the other hand, it provided a heterogenic population, including men and women of a variety of modalities.

Furthermore, it should be pointed out that the main results described have been carried out based on the results obtained and selected, under our own eligibility criteria, using valid and reliable tools to classify the different potential risk injury factors.

## 5. Conclusions

From the detection of asymmetries, using the OB to measure, in the medial, superolateral and inferolateral directions, mobility and balance can be assessed, allowing physical or physiological misalignments between different body segments to be observed, which can negatively influence the athlete’s performance or increase the risk of injury.

Likewise, OB is shown as an assessment tool which allows detection of asymmetries in the upper limb, being necessary on the part of the athletes, of a neuromuscular work, especially in the upper body, with a specific training which allows the reduction in asymmetries and the improvement of mobility and balance.

The results obtained in this project, highlighted the importance of a personal and individual assessment of the athlete, in each of the stages in which the season is divided, which will help in the prevention of injuries and in the individualization of training.

## Figures and Tables

**Table 1:** Characteristics of the participants in the study.

	*n*	Mean	Standard Deviation
Age (years)	252	23.33	8.96
Height (cm)	252	178.63	11.12
Body mass (kg)	252	80.28	17.61
BMI	252	24.88	4.58
Experience in sport (years)	252	12.52	6.28

**Table 2:** Age of participants, gender of participants, sport and type of sport.

Age	*n*	Percent
Younger than 18	65	25.8
18–35	173	68.7
Older than 35	14	5.6
Total	252	100.0
Gender	*n*	Percent
Men	210	83.3
Women	42	16.7
Total	252	100.0
Sport	*n*	Percent
Rugby	91	36.1
Athletics	22	8.7
Football	48	19.0
Swimming	8	3.2
Handball	39	15.5
Triathlon	4	1.6
Baskeball	10	4.0
Hockey	9	3.6
Badminton	7	2.8
Volleyball	14	5.6
Total	252	100.0
Sport	*n*	Percent
Rugby	91	36.1
Athletics	22	8.7
Football	48	19.0
Swimming	8	3.2
Handball	39	15.5
Triathlon	4	1.6
Baskeball	10	4.0
Hockey	9	3.6
Badminton	7	2.8
Volleyball	14	5.6
Total	252	100.0
Type of sport	*n*	Percent
Endurance	34	13.5
Intermittent	218	86.5
Total	252	100.0

**Table 3:** OB values in the medial direction of the left arm.

	Mean	Std. Deviation	F	Sig
OB Med L M17 (cm)	71.02	7.15	5.820	0.012
OB Med L S17 (cm)	68.49	9.15		
OB Med L F18 (cm)	69.50	8.66		
OB Med L M18 (cm)	65.03	7.66		

OB: OctoBalance test; Med: Medial; L: left arm; M17: May 2017; S17: September 2017; F18: February 2018; M18: May 2018.

**Table 4:** OB values in the medial direction of the right arm.

	Mean	Std. Deviation	F	Sig
OB Med R M17 (cm)	71.19	8.07	2.273	0.137
OB Med R S17 (cm)	69.80	10.38		
OB Med R F18 (cm)	69.83	8.78		
OB Med R M18 (cm)	66.03	9.55		

OB: OctoBalance test; Med: Medial; R: right arm; M17: May 2017; S17: September 2017; F18: February 2018; M18: May 2018.

**Table 5:** OB values in the superolateral direction of the right arm.

	Mean	Std. Deviation	F	Sig
OB Sup R M17 (cm)	37.27	7.93	2.416	0.117
OB Sup R S17 (cm)	32.86	9.40		
OB Sup R F18 (cm)	54.24	85.77		
OB Sup R M18 (cm)	38.60	10.69		

OB: OctoBalance test; Sup: Superolateral; R: right arm; M17: May 2017; S17: September 2017; F18: February 2018; M18: May 2018.

**Table 6:** OB values in the superolateral direction of the left arm.

	Mean	Std. Deviation	F	Sig
Ob Sup L M17 (cm)	35.27	7.09	2.999	0.077
Ob Sup L S17 (cm)	33.29	8.41		
Ob Sup L F18 (cm)	32.40	7.80		
Ob Sup L M18 (cm)	39.63	12.06		

OB: OctoBalance test; Sup: Superolateral; L: left arm; M17: May 2017; S17: September 2017; F18: February 2018; M18: May 2018.

**Table 7:** OB values in the inferolateral direction of the right arm.

	Mean	Std. Deviation	F	Sig
Ob Inf R M17 (cm)	39.86	9.52	2.051	0.160
Ob Inf R S17 (cm)	47.41	10.96		
Ob Inf R F18 (cm)	44.42	8.92		
Ob Inf R M18 (cm)	43.41	6.07		

OB: OctoBalance test; Inf: Inferolateral; R: right arm; M17: May 2017; S17: September 2017; F18: February 2018; M18: May 2018.

**Table 8:** OB values in the inferolateral direction of the left arm.

	Mean	Std. Deviation	F	Sig
Ob Inf L M17 (cm)	45.30	6.31	1.224	0.347
Ob Inf L S17 (cm)	44.45	8.51		
Ob Inf L F18 (cm)	41.50	9.09		
Ob Inf L M18 (cm)	43.82	7.85		

OB: OctoBalance test; Inf: Inferolateral; L: left arm; M17: May 2017; S17: September 2017; F18: February 2018; M18: May 2018.

## Data Availability

The data presented in this study are available on request from the corresponding author. The data are not publicly available due to privacy.
